# A Rare Case of Inguinal Hernia with Complete Bladder Herniation

**DOI:** 10.1155/2017/4658169

**Published:** 2017-10-31

**Authors:** Ayaaz Habib

**Affiliations:** Nottingham University Hospitals NHS Trust, Queen's Medical Centre, Derby Road, Nottingham NG7 2UH, UK

## Abstract

Involvement of the bladder in inguinal hernias is rare and occurs in less than 5% of the cases. The diagnosis and management of this condition may present a challenge to the surgeon. We present a case of an elderly gentleman who presented with a large left-sided inguinoscrotal hernia causing an obstructive uropathy which was surgically repaired. The patient made a quick postoperative recovery with complete resolution of renal function.

## 1. Introduction

Inguinal hernias are common with the lifetime risk of 27% in men and 3% in women [1]. It has been estimated that approximately 20 million inguinal hernia repairs are performed annually worldwide [2]. Herniation of the bladder in an inguinal hernia occurs rarely and represents 0.5–3% of lower abdominal hernias. They are more predominant in men aged between 50 and 70 [3].

In cases where the entire bladder herniates into the scrotum, the patient completes two-stage urination (manual compression of the scrotum to empty the bladder) [4]. The majority of patients with bladder hernias are asymptomatic, and diagnosis is made intraoperatively. Inguinoscrotal bladder hernias are associated with significant urological complications such as obstructive uropathy, urinary tract infections, and bladder infarctions [5]. The diagnosis and intraoperative management may be challenging to the surgeon. We hereby report an unusual case of an elderly gentleman who presented with a massive left-sided inguinoscrotal hernia with complete bladder involvement presenting as declining renal function and bilateral hydronephrosis.

## 2. Case Presentation

A 78-year-old retired solicitor with a previous history of left-sided inguinal hernia presented to the emergency department due to a deterioration in renal function. The hernia was first diagnosed 5 months previously, and it was decided to keep the patient under watchful waiting as he was asymptomatic. There was no suspicion of bladder outlet obstruction previously. On this admission, he complained of left-sided groin pain. He denied abdominal pain. There was no history of nausea or vomiting. He had a good appetite, and there was no weight loss reported. His bowels were functioning normally, and he had a long-term urinary catheter in situ. The patient reported having to manually compress the scrotum in order to empty the bladder. There were no other urinary symptoms. His past medical history included hypertension and TURP (transurethral resection of the prostate) in 1998 and a redo TURP in 2009 for prostatic hypertrophy from which he was asymptomatic. His medications included Amlodipine 5 mg once daily and Tamsulosin 400 µg once daily. He had no known drug allergies. He lived in a residential home and was independent. He was an ex-smoker with a 10 pack-year history and only consumed alcohol socially.

On clinical examination, his vital signs were normal. Cardiovascular and respiratory systems were unremarkable. A large left-sided inguinoscrotal hernia was obvious on inspection which was mildly tender on palpation. It was irreducible. His abdomen was otherwise soft with normal bowel sounds. He had an indwelling catheter draining clear urine. A timeline of the case is given in Table 1.

**Figure 1:**
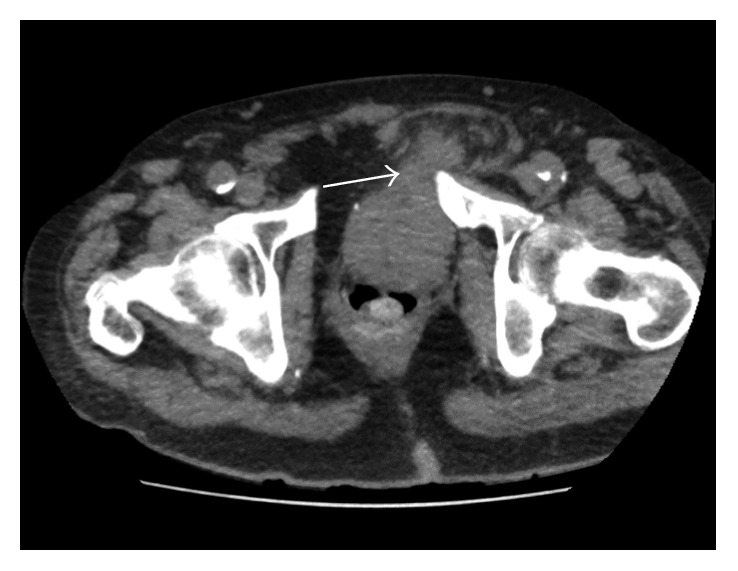
Axial CT scan section showing herniation of the bladder into the left inguinal region.

**Figure 2:**
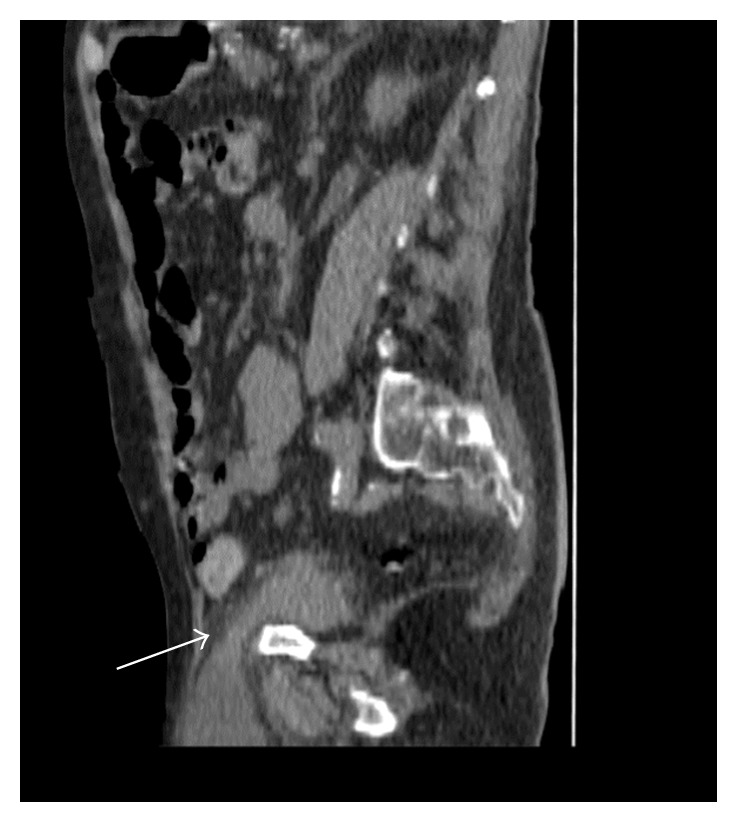
Sagittal CT section showing bladder herniation.

Initial investigations showed a haemoglobin of 11 g/dL (13–18 g/dL), white cell count of 14 × 10^9^/L (4–11 × d10^9^/L), and platelets of 290 × 10^9^/L (150–350 × 10^9^/L). His renal function showed the following: Na^+^ of 142 mmol/L (135–145 mmol/L); K^+^ of 4.6 mmol/L (3.6–5.1 mmol/L); urea of 14.4 mmol/L (2.5–6.6 mmol/L); creatinine of 208 µmol/L (60–120 µmol/L), and an EGFR (estimated glomerular filtration rate) of 25 mL/min/1.73 m^2^ (baseline 75 mL/min/1.73 m^2^). He was initially managed with intravenous fluids and analgesia whilst awaiting an urgent CT scan of the abdomen and pelvis (Figures 1 and 2). He also had an ultrasound scan of the kidneys preoperatively.

**Figure 3:**
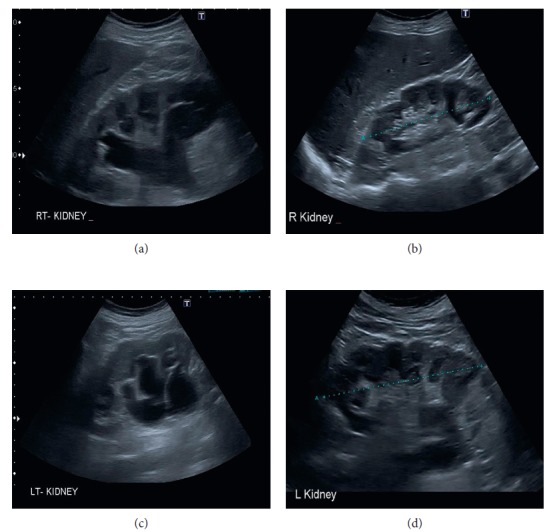
Preoperative ultrasound scan of the right kidney showing hydronephrosis (a) and resolution postoperatively (b). Hydronephrosis of left kidney (c) and postoperative resolution (d). Cortical cysts are seen.

Following the CT scan, he was taken to the theatre within 4 days for hernia repair. Prior to this, he was treated with intravenous fluids with a strict record of fluid balance, regular review by the renal physicians (who advised input to match output + 30 mL per hour), analgesia, and half a dose (20 mg) of prophylactic enoxaparin as part of venous thromboembolism (VTE) risk reduction given the poor renal function. Intraoperative findings revealed a direct left inguinal hernia with complete herniation of bladder into the scrotum with the catheter balloon. The bladder appeared healthy with no signs of injury. This was restored to its normal anatomical position. The hernia was repaired with a biologic mesh (EGIS® porcine dermal implant, 10 × 10 cm) by the Lichtenstein technique. He made an uneventful recovery postoperatively with improvement of his renal function. He was discharged to the residential home on day 7. A renal ultrasound scan 6 weeks after the procedure showed resolution of the hydronephrosis and improvement of renal function back to baseline (Figure 3). He is under regular urology follow-up.

## 3. Discussion

This was a case of a large left-sided inguinoscrotal hernia with complete bladder herniation presenting as acute renal failure. This was repaired surgically. Inguinal bladder hernia was first described by Levine in 1951 as a scrotal cystocele, which is a rare clinical finding [6]. This condition has been reported extensively in literature, primarily in the form of case reports and case series [5, 7–11]. Inguinal bladder hernias mostly occur in the elderly, and associated risk factors include obesity, chronic urinary obstruction, and a weak pelvic musculature [12–14]. Benign prostatic hypertrophy (BPH), hydronephrosis with or without acute kidney injury, vesicoureteric reflux, urinary tract infections, bladder necrosis, and scrotal abscesses are pathologies associated with inguinal bladder hernias [5]. In our case, the patient presented with bilateral hydronephrosis indicating ureteric involvement due to compression within the hernial sac. In the context of bladder hernias, obstructive renal failure due to ureteric involvement is also a rare finding [15].

Patients with bladder hernias usually present with lower urinary tract symptoms. In more advanced cases, two-stage urination is seen where the first stage is spontaneous and the second stage is facilitated by manual scrotal compression [4, 12]. However, patients can also be asymptomatic. Imaging modalities include CT scanning, intravenous urogram, and cystography. A case series has demonstrated the success of all three imaging techniques [5]. Ultrasonography can be used to detect the presence of hydronephrosis and to differentiate the bladder from other intrascrotal conditions such as a hydrocele, epididymal cysts, and abscesses [16]. Given the advanced nature of the case presented above, a CT scan was sufficient to make a prompt diagnosis and plan the surgical approach.

The standard treatment of inguinal bladder hernias is surgical repair (herniorrhaphy) [5, 10]. In the past, surgeons have resected the herniated portions of the bladder where the hernia was found to be massive [5]. However, current recommendations are to perform resection where this is evidence of bladder wall necrosis, herniated bladder diverticulum, a tight hernia neck, or a bladder tumour [15, 17]. Fortunately, our patient did not show any of these signs. Repair of the hernia can be performed with the use of a mesh to prevent recurrence. Some patients may also opt for conservative management of watchful waiting or intermittent self-catheterization [4]. We would recommend these options only for asymptomatic or minimally symptomatic patients. In our case, surgery was the mainstay of management given the presence of advanced disease and renal failure. The main point is that this entity is rare yet associated with significant complications. Furthermore, this condition is a surgical challenge, and preoperative imaging is useful in planning the approach and anticipating difficulties. This case report describes a rare and unusual case of a bladder hernia. The risk factors, diagnosis, complications, and management strategies are discussed. Limitations include the retrospective nature of this study and the lack of the ability to generalize.

## 4. Conclusion

Inguinal bladder hernias are rare. They are often difficult to diagnose and remain a surgical challenge. It is important to suspect the diagnosis in a patient with a known history of an inguinal hernia where renal function is acutely compromised. Preoperative imaging is essential to prevent iatrogenic injury and complications associated with this condition. As surgical repair is the mainstay of management, it is important for the general surgeon to have a sound understanding of this condition.

## Figures and Tables

**Table 1:** Case timeline.

Past medical history—benign prostatic hypertrophy and hypertension.		
The left-sided inguinal hernia was first diagnosed on 11 October 2016. The patient was put under watchful waiting as he was asymptomatic. A routine blood test by the general practitioner in February 2017 showed severely compromised renal function compared with baseline which prompted a referral to the emergency department for further evaluation.		
Current illness	Left-sided groin pain and two-stage urination	9/2/17
	Left-sided inguinoscrotal hernia and renal failure	

Physical examination	Large left-sided inguinoscrotal hernia with minimal tenderness. Abdomen soft and nontender. Bowel sounds normal	9/2/17

Diagnostic evaluation	Blood tests (see text)	9/2/17
	CT scan (Figures 1 and 2)	
	Renal ultrasound (Figure 3)	

Diagnosis	Large left-sided inguinoscrotal hernia with bladder herniation and bilateral hydronephrosis	9/2/17

Initial treatment	Intravenous fluids, analgesia, fluid balance, and renal physician input	9/2/17–12/2/17

Final treatment	Surgical repair of hernia by the Lichtenstein technique	13/2/17

Follow-up	Renal ultrasound 6 weeks postoperatively showing resolution of hydronephrosis	25/4/17
